# Evaluation of the Antibacterial Effects and Mechanism of Action of Protocatechualdehyde against *Ralstonia solanacearum*

**DOI:** 10.3390/molecules21060754

**Published:** 2016-06-09

**Authors:** Shili Li, Yanmei Yu, Juanni Chen, Bing Guo, Liang Yang, Wei Ding

**Affiliations:** Laboratory of Natural Products Pesticides, College of Plant Protection, Southwest University, Chongqing 400715, China; lsl203lst@163.com (S.L.); trcmei@126.com (Y.Y.); chenhuanni521@126.com (J.C.); guobing425@163.com (B.G.); ylwzling@163.com (L.Y.)

**Keywords:** *Ralstonia solanacearum*, protocatechualdehyde, antibacterial activity, biofilm formation, bacterial wilt

## Abstract

Protocatechualdehyde (PCA) is an important plant-derived natural product that has been associated with a wide variety of biological activities and has been widely used in medicine as an antioxidant, anti-aging and an anti-inflammatory agent. However, fewer reports concerning its antibacterial effects on plant-pathogenic bacteria exist. Therefore, in this study, protocatechualdehyde was evaluated for its antibacterial activity against plant pathogens along with the mechanism of its antibacterial action. PCA at 40 μg/mL was highly active against *R. solanacearum* and significantly inhibited its growth. The minimum bactericidal concentration and minimum inhibitory concentration values for PCA were 40 μg/mL and 20 μg/mL, respectively. Further investigation of the mechanism of action of PCA via transmission electron microscopy and biological assays indicated that the destruction of the cell structure, the shapes and the inhibition of biofilm formation were important. In addition, the application of PCA effectively reduced the incidence of bacterial wilt on tobacco under greenhouse conditions, and the control efficiency was as high as 92.01% at nine days after inoculation. Taken together, these findings suggest that PCA exhibits strong antibacterial activity against *R. solanacearum* and has the potential to be applied as an effective antibacterial agent for controlling bacterial wilt caused by *R. solanacearum*.

## 1. Introduction

*Ralstonia solanacearum* is a rod-shaped Gram-negative plant-pathogenic bacterium that causes bacterial wilt disease [1,2,3]. This soil-borne bacterium can infect more than 200 plant species, mainly in the *Solanaceae* and *Musaceae* families [4]. This soil-borne pathogen can also cause typical wilting symptoms by colonization, invasion, survival and growth in the root system and xylem tissue via wounds or natural openings [5,6]. During the development of disease, *R. solanacearum* cells secrete several virulence factors, including an extracellular polysaccharide, several plant cell wall–degrading enzymes and some type III–secreted effectors [7,8,9,10,11]. The pathogen is widely distributed in tropical, subtropical and some temperate regions and affects significant economic crops, such as tomato, banana, potato, and tobacco. The bacterium can be free-living in soil or in water as a saprophyte after destroying the host [12]. Therefore, *R. solanacearum* is one of the most destructive pathogens of many economically important plants [3].

To control bacterial wilt, agrochemicals, such as copper derivatives, antibiotics and quaternary ammonium compounds, are conventionally used for crop protection [13,14]. However, the application of these traditional agrochemicals has been proven not very effective in controlling this soil-borne bacterium. At the same time, the overuse of bactericides can cause severe side effects, especially the production of resistant strains and environmental pollution [15,16]. Agricultural controls, including resistant cultivars, tillage management, pathogen-free transplants and crop rotation are widely used in prevention research. However, because *R. solanacearum* can remain in the soil for a long time outside of the host plant, few efforts involving agricultural management have been made to control *R. solanacearum* once a bacterial wilt outbreak has occurred [17,18]. Thus, there is an immediate need for the development of a new alternative agent or potent anti–*R. solanacearum* compounds to effectively control this bacterium.

Botanical substances contain abundant bactericidally active materials, and these materials have activity against a broad spectrum of bacteria, are easily decomposed, are unlikely to pollute the environment, have no significant toxicity to vertebrates and the host plant, and have a low cost of renewal and other advantages [4,19]. Several studies have demonstrated that the use of botanical compounds to prevent *R. solanacearum* infection may be effective. Methyl gallate (MG) from *Toxicodendron sylvestre* was shown to significantly inhibit *R. solanacearum* growth *in vitro* and *in planta* [20,21], and it was shown to be able to damage the cell wall structure, inhibit protein synthesis and succinate dehydrogenase (SDH) activity in the pathogen [22]. Similarly, the antibacterial activity of Lansiumamide B on *R. solanacearum* and its control efficiency in potting medium were evaluated by *in vitro* assays [4]. Earlier experiments demonstrated that thymol, palmarosa, lemongrass, and eucalyptus oils could reduce *R. solanacearum* populations in heavily infested potting medium [23,24] by inducing changes in the cell structure at the sub-cellular level and caused a significant reduction of many physiological metabolites [25]. Others substances, including active components of gallnuts [26], essential oils of cinnamon, thyme, lavender, eucalyptus [14], and an *Allium fistulosum* extract [27], have been reported to have antibacterial activity against *R. solanacearum*. However, most current research is focused on botanical compounds for the control of *R. solanacearum* in *in vitro* assays or in potted plants, and few plant compounds have been used to control plant bacterial wilt *in planta* until now. The use of botanically active materials for the development of new, environmentally friendly pesticides will become an inevitable trend [28].

Protocatechualdehyde (protocatechnic aldehyde, PCA) is a natural polyphenol compound that is isolated from the roots of traditional Chinese medicine radix *Salviae Miltiorrhizae* [29,30], and previous studies have shown that PCA has pharmacological properties, including antioxidant, cardiovascular and anti-inflammatory activities [31]. Although PCA has been reported to have broad antibacterial anti-inflammatory effects, its antibacterial activity has not been documented. Therefore, in this study, we evaluated the antibacterial potential of PCA against soil-borne pathogens *in vitro* and *in vivo*, as well as its mechanism of action.

## 2. Results

### 2.1. The Minimum Inhibitory Concentration and Minimum Bactericidal Concentration Values of PCA on R. solanacearum

**Figure 1:**
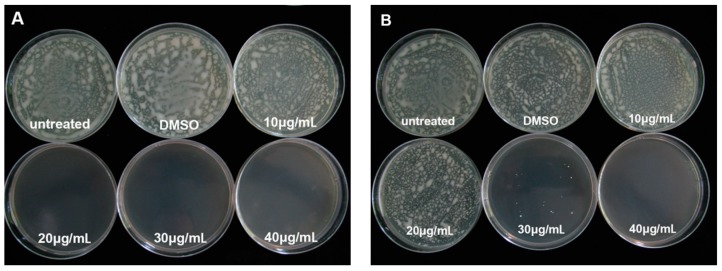
Effect of PCA on the growth of *R. solanacearum* after cultured for 48 h (**A**) and 96 h (**B**).

The minimum inhibitory concentration (MIC) and minimum bactericidal concentration (MBC) of PCA against *R. solanacearum* were measured by using the serial two-fold agar dilution method. The MIC and MBC value against *R. solanacearum* were 20 μg/mL and 40 μg/mL, respectively. It was able to completely inhibit *R. solanacearum* at a concentrations of 40 μg/mL on agar media (Figure 1). However, the solvent dimethylsulfoxide (DMSO) had no effect on the growth of *R. solanacearum* on plates at 48 h and 96 h.

### 2.2. R. solanacearum Growth Curves with PCA Treatment

**Figure 2:**
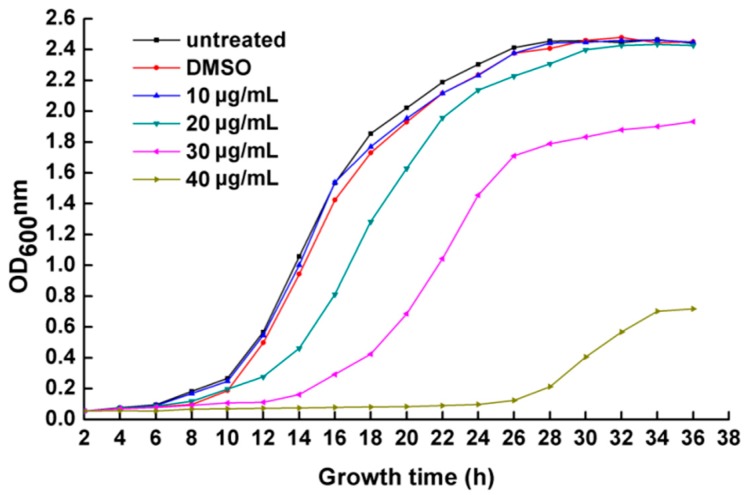
OD growth curves of *R. solanacearum* in the presence of different PCA concentrations at 30 °C. Untreated bacteria were treated with sterile water. Bacteria were also treated with DMSO alone at a final concentration of 0.1%. The OD value of each treatment was the average of three replicates.

In this study, the antibacterial activity of PCA was estimated by growth curves using the turbidimeter test method. The cells were treated with PCA concentrations of 10, 20, 30 and 40 μg/mL, and PCA significantly inhibited the growth of the bacteria at 30 and 40 μg/mL treatment; a PCA concentration of 40 μg/mL almost completely stopped *R.* *solanacearum* growth after 26 h incubation (Figure 2).

### 2.3. Morphology Characterization of R. solanacearum by SEM

**Figure 3:**
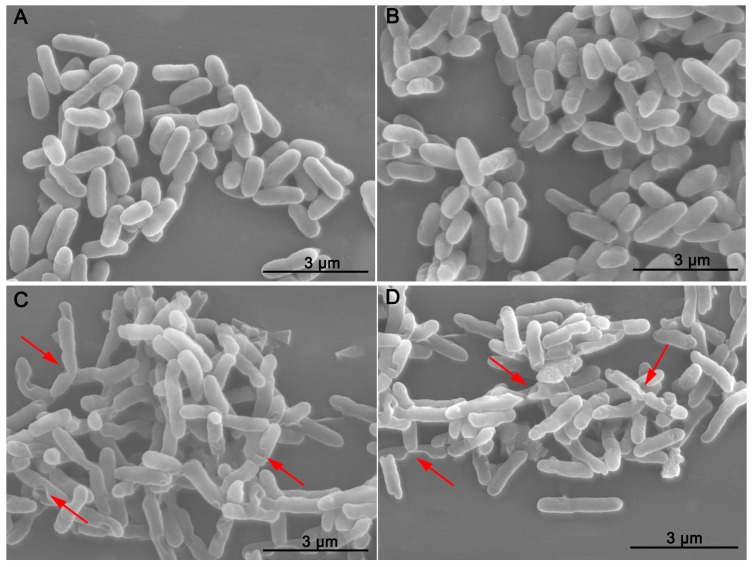
SEM images of *R. solanacearum* cells treated with PCA at different concentrations. (**A**) Untreated cells; (**B**) Cells treated with DMSO alone at a final concentration of 1%; (**C**) Cells treated with 30 μg/mL PCA; (**D**) Cells treated with 40 μg/mL PCA. Cells were incubated for 12 h with shaking at 180 rpm at 30 °C.

**Figure 4:**
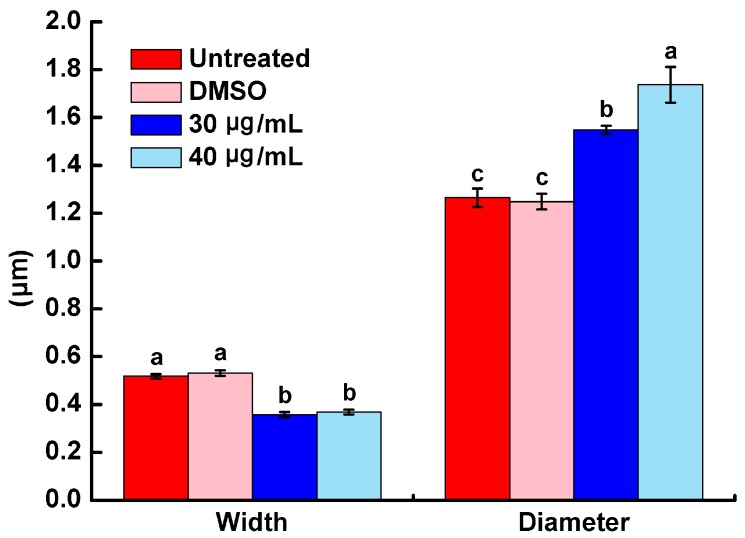
Effects of different treatments on width and diameter of *R. solanacearum* cells. The mean value of width and diameter was calculated based on scale bars of SEM images. The error bars indicate the standard error of the mean from 100 cells. Lower case letters indicate significant differences according to Duncan’s test (*p* < 0.05).

SEM images of the morphology of *R. solanacearum* after inoculation with PCA concentrations of 30 and 40 μg/mL were taken. Under our experimental conditions, the untreated bacteria (Figure 3A) and 0.1% DMSO–treated (Figure 3B) bacteria showed typical *R. solanacearum* morphology—a short rod shape with the integrity of the membrane structure intact and a smooth texture; moreover, no significant difference between these treatments was evident. In contrast, PCA treatment at both concentrations obviously destroyed the surface structure of the bacterial cells and shapes (Figure 3C,D and Figure 4). The shape of the treated cells was longer than the untreated cells and their cellular integrity was lost at a concentration of 30 and 40 μg/mL (Figure 3C and Figure 4). Additionally, the widths of treated cells were significantly decreased compared to the control groups (Figure 4). In addition, the cells treated with 40 μg/mL PCA showed greater damage than those treated with 30 μg/mL PCA (Figure 3D and Figure 4). These results suggested that PCA could damage the cell wall of *R. solanacearum* and that the damaging effect was greater at a higher concentration.

### 2.4. Effect of PCA on Biofilm Formation in R. solanacearum

**Figure 5:**
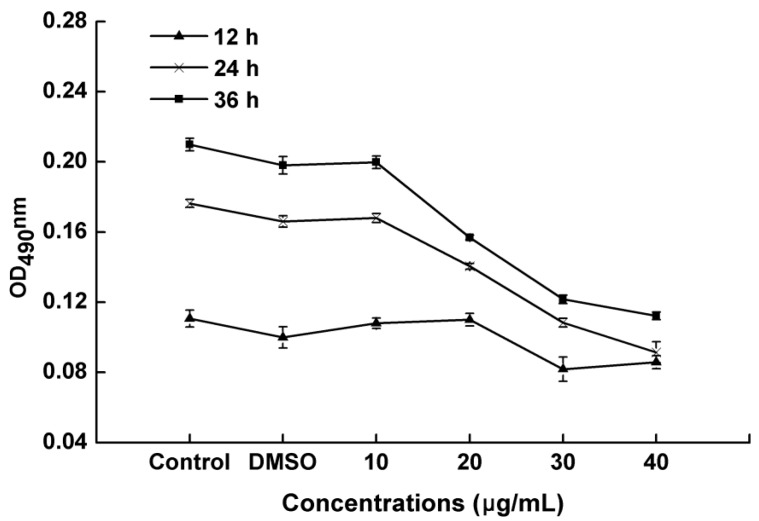
Effects of different concentrations (10, 20, 30 and 40 μg/mL) of PCA on biofilm formation in *R. solanacearum*. Cells were treated with DMSO alone at a final concentration of 1% (DMSO treatment), sterile water (Untreated) and PCA at a final concentration of 10, 20, 30 and 40 μg/mL (PCA treatments). Experiments were incubated for 12, 24 and 36 h at 30 °C without shaking.

**Figure 6:**
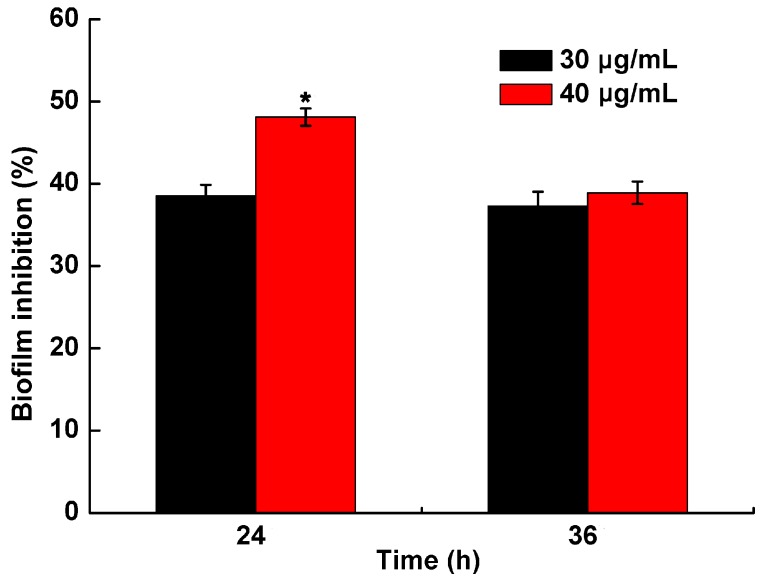
Inhibition effect of PCA on biofilm formation of *R. solanacearum*. Biofilm inhibition (%) was quantified after treatment with concentrations of 30 and 40 μg/mL at 24 and 36 h in the 96-well plates. The symbol indicate significant differences according to independent-samples *T* test (* indicated *p* < 0.01).

After treatment with PCA concentrations of 10, 20, 30 and 40 μg/mL, the biofilm formation of *R. solanacearum* was determined after 12, 24 and 36 h. The biomass in all treatments increased with time from 12 h to 36 h (Figure 5). Biofilm formation was significantly greater in the control and DMSO (without PCA) groups than in the treatment groups ranging from 0.11 to 0.21 and 0.10 to 0.20, respectively. Furthermore, the bioactivity of PCA activity was concentration-dependent, and the biofilm formation was gradually suppressed by PCA as the concentrations increased (Figure 5). A low concentration of 10 μg/mL did not inhibit biofilm formation, but 30 and 40 μg/mL were found to notably inhibit biofilm formation. Compared with the control, biofilm formation after the 40 μg/mL treatment significantly reduced biofilm formation by 48.11% and 38.90%, and by 38.56% and 37.27% after the 30 μg/mL treatment, at 24 and 36 h, respectively (Figure 6). The results showed that PCA exhibited a strongly inhibitory on *R.* *solanacearum* biofilm formation.

### 2.5. Assessment of R. solanacearum Swarming Motility in the Presence of PCA

**Figure 7:**
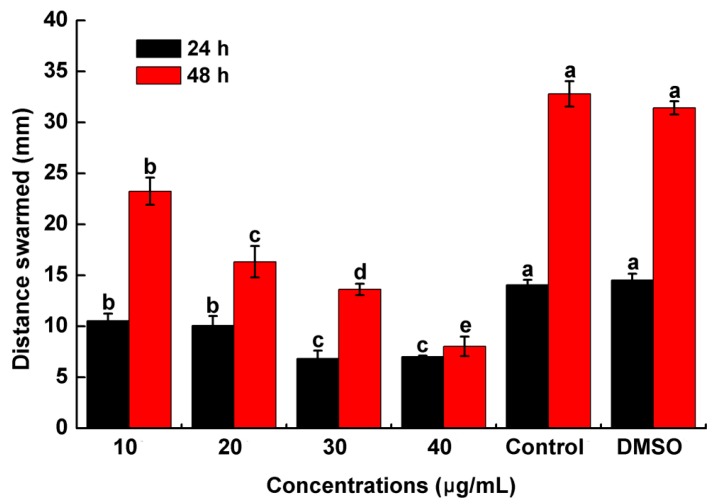
Effects of PCA at different concentrations on *R. solanacearum* swarming in swarming medium. The swarming diameter was measured in both the vertical and horizontal direction on each plate after incubation for 24 h and 48 h at 28 °C. The mean value in both directions was calculated. The diameter represents the average of triplicate plates. The assays were independently repeated three times. The error bars indicate the standard error of the mean from three replicates. Lower case letters indicate significant differences according to Duncan’s test (*p* < 0.05).

Motility was considered to be closely and positively related with biofilm formation. The previous experiment demonstrated that PCA could obviously suppress biofilm formation. We investigated whether PCA could affect the motility of *R. solanacearum* in a petri dish. The results indicated that PCA could significantly inhibit swarming motility at concentrations ranging from 10 to 40 μg/mL after 24 and 48 h (Figure 7). At 24 h, the diameter of the migration zone was decreased by 2.12- and 2.07-fold compared with the control. Moreover, the inhibitory effect of PCA on the swarming motility after 48 h was more evident and the diameter of the migration zone was reduced by 4.08-fold. This experiment indicated that PCA potently exerted an obvious inhibitory effect on the swarming motility of *R. solanacearum* in a dose-dependent manner.

### 2.6. The Pathogenicity of R. solanacearum in a Greenhouse Treated with PCA

**Figure 8:**
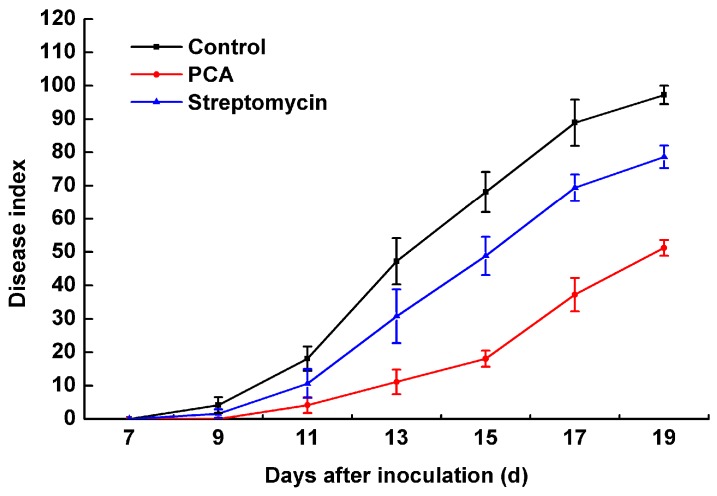
Disease index of bacterial wilt of tobacco with PCA treatment. Unwounded tobacco plants were soil-soak inoculated at concentrations of 5 mL (1 × 10^8^ cfu/mL) and inoculated at 28 ± 1 °C after treatment with 40 μg/mL PCA. Untreated plants were treated with sterile water. The mean of three replications and the data were from a single representative and reproduced several times. The disease index was expressed as the mean ± SE.

**Figure 9:**
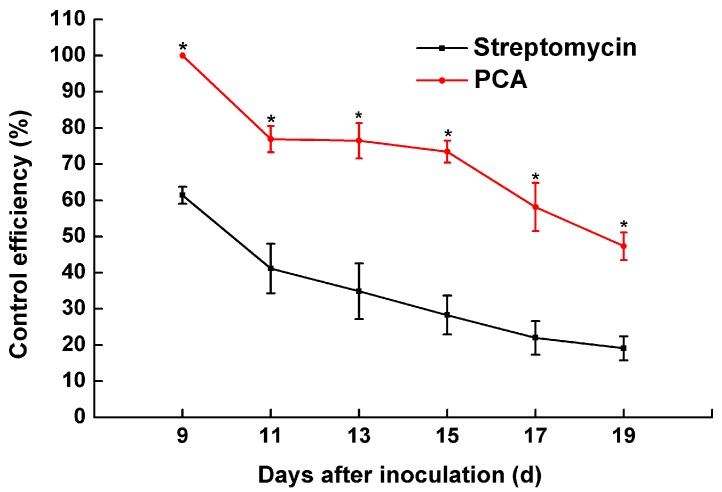
The control effect of PCA on tobacco inoculated with *R. solanacearum*. The bars indicate the standard error of the mean of three replicates. * indicates a significant difference between treatments (independent-samples *T* test, *p* < 0.05).

PCA at a concentration of 40 μg/mL had the most effective *in vitro* antibacterial activity, so we selected this concentration for disease assessment in a pot experiment. Beginning at seven days after inoculation, the incidence of disease was assessed every two days from seven to 19 days. Typical bacterial wilt symptoms were observed nine days after the tobacco plants were inoculated with *R. solanacearum* (Figure 8). A significant effect of PCA treatment was shown for the disease index of bacterial wilt. The disease indices of PCA and streptomycin treatments on tobacco bacterial wilt were 18.06 and 51.22 and 48.82 and 78.65, respectively, at 15 and 19 days after inoculation. However, the disease index of the control reached 68.06 and 97.22 at 15 and 19 days, respectively (Figure 8). Moreover, the control efficiency of the PCA and the streptomycin treatments were 92.01% and 47.31% and 61.39% and 19.10% at nine and 19 days after inoculation (Figure 9), respectively. A concentration of 40 μg/mL of PCA showed extremely significant effects on the control of tobacco bacterial wilt of over 30.62% and 28.21% compared to streptomycin at nine and 19 days, respectively.

## 3. Discussion

Bacterial wilt of tobacco is a devastating disease worldwide and its threat is constantly increasing. For the last few years, traditional chemosynthesis pesticides have been used to control soil-borne diseases caused by *R.* *solanacearum*. However, many chemical pesticides pollute the environment and lead to the accumulation of residues in crops and soil. Therefore, in pesticide science, it is essential to search for new natural antibacterial agents that are highly efficient and environmentally friendly [32]. The application of natural products isolated from plants and biological agents has been shown to be effective and environmentally friendly against many plant pathogens [33]. In nature, plants contain a large variety of natural products, many of which have evolved to have antimicrobial activity against pathogens that cause bacterial and fungal diseases, such as flavonoids [34], phenols [35], sesqrepenes [36], alkaloids [37], and coumarins [38]. PCA is a water-soluble antioxidant phenolic aldehyde extracted from the roots of *Salvia miltiorrhiza*, and it has been widely used in many fields of medicine and biological research [39,40]. However, its potential bactericidal activity against soil-borne pathogens has not been intensively investigated. Therefore, in this research, the antibacterial potentials of PCA against *R.* *solanacearum* and the mechanism of action were investigated.

PCA is an important biologically active component of some traditional Chinese medicines. Previous studies have indicated that PCA has antioxidant activity, antibacterial activity [41], anticancer activity [42], anti-aging activity [43], and anti-inflammatory activity [44]. Furthermore, PCA is a widely distributed, naturally occurring phenolic acid and has structural similarity with caffeic acid, syringic acid and gallic acid [45]. Therefore, we speculated that these compounds had strongly similar biological functions. Polyphenols are secondary metabolites that are found ubiquitously in many higher plants and play important roles in defending against plant pathogens by suppressing microbial virulence factors, such as inhibiting biofilm formation and reducing adhesion to the host [35]. Lee *et al.* reported that tea catechins showed a significant antipathogenic effect against *Escherichia coli* O157:H7 by suppressing biofilm formation and swarming motility [46]. Those results were consistent with the results of this study in which PCA inhibited biofilm formation and the swarming ability of *R. solanacearum* (Figure 5, Figure 6 and Figure 7).

PCA, caffeic acid and gallic acid are all polyphenols that have potential antibacterial activity for food and plant diseases. Moreover, these compounds all have a similar chemical structure, consisting of a benzene ring with two or three hydroxyl groups. Their structural similarity and diversity may be involved in determining their anti-tumor, anti-bacterial, and anti-proliferative bioactivity. Farag *et al*. demonstrated that gallic acid extracted from *Acacia arabica* and *Punica granatum* displayed significant antimicrobial activity against *R. solanacearum* (MIC values 0.5–9 mg/mL) [47]. Methyl gallate also showed a strong inhibitory effect on *R. solanacearum* at an IC_50_ of 8.3 mg/L and MIC of 20 mg/L. A disease control trial *in planta* indicated that methyl gallate could effectively control tomato bacterial wilt [20]. In addition, gallic acid possessed a high antifungal activity against *Fusarium solani* via the degradation of fungal cell walls [48]. In addition, Zhao *et al.* demonstrated that flavonoids isolated from the Chinese medicinal plant *Dalbergia odorifera* exhibited a stronger inhibitory activity against *R. solanacearum* [16]. In this study, our findings indicated that PCA exhibited a strong inhibitory effect on the growth of *R.* *solanacearum* *in vitro* (Figure 1 and Figure 2), and also could control tobacco bacterial wilt effectively *in planta* (Figure 9).

The MIC of an antibacterial agent is the lowest concentration required to simply inhibit the growth of bacteria. The MBC is the minimal concentration of an agent that kills a particular bacterium. An antibacterial agent is usually regarded as bacterial if the MBC is not more than four times the MIC [49]. The result reported in this study showed that the MBC (40 μg/mL) was two times the MIC (20 μg/mL) for PCA, which means it does meet this general rule of thumb to be labeled as an antibiotic. However, it should also be noted that some antibiotics caused an aggregation effect of bacterial cells which could have large impacts on the MIC and MBC calculations. Previous studies showed that some flavonoids induce bacterial aggregation [50]. Then further findings demonstrated that the flavonol galangin caused *Staphylococcus aureus* cells to clump together, and this aggregation effect led to the decreases in bacterial numbers detected in the time-kill assay and the MBC was determined [51]. The decreases in MBC assays may have been caused by bacterial aggregation rather than cell death. In this report, the calculated MIC and MBC of PCA were measured by using the agar dilution technique. The time-kill assay conducted in the present study indicated that 40 μg/mL of PCA could strongly inhibit the growth of the bacteria, and this result was consistent with that measured by the agar dilution assay. Therefore, it could be speculated that PCA could not cause the aggregation of *R.* *solanacearum* cells.

In this report, we have shown that PCA could significantly decrease the incidence of tobacco bacterial wilt at concentrations greater than 40 μg/mL in a greenhouse experiment. Although PCA has shown potential as an effective bactericide for the control of bacterial wilt, it is not known whether the control efficiency can be sustained during the tobacco growth cycle in the field. Further research is required to analyze the *Ralstonia* populations’ dynamics, and to explore the optimal application and duration of protection. In addition, a new form of plant-type antibiotic or a new antibacterial drug could be developed via minor modifications based on the structure of PCA. New studies for the further verification of the antibacterial mechanism of PCA against *R. solanacearum* at the physiological and molecular levels need to be performed. These studies would provide a scientific and theoretical basis for developing novel compounds and improving their antibacterial effects.

## 4. Materials and Methods

### 4.1. Chemicals and Bacterial Strains

PCA (HPLC ≥ 98%) used in this study was purchased from the Shanghai Yuanye Bio-Technology Co., Ltd. (Shanghai, China). PCA was dispersed in 20% dimethyl sulfoxide (DMSO) at a concentration of 20 mg/mL and diluted in sterile distilled water (ddH_2_O) to the desired concentration. *R. solanacearum* (phylotype І, race1, biovar 3) was used throughout the study [52].

### 4.2. Determination of the MIC and the MBC

The minimum inhibitory concentration (MIC) and the minimum bactericidal concentration (MBC) of PCA was calculated by agar dilution assay at different concentrations (10, 20, 30, 40 μg/mL) in petri dishes. One hundred microliters of a *R. solanacearum* suspension adjusted to 1 × 10^5^ cfu/mL was spread directly onto each antibiotic-containing agar dilution plate. Plates with 1% DMSO but without PCA and without any additives were used as controls. The plates were incubated at 30 ± 1 °C. The MIC was determined as the lowest concentration at which no colony formation was observed after cultured 48 h. The MBC was defined as the lowest concentration of PCA that prevented the growth of bacteria after cultured 96 h. All assays were independently repeated at least three times.

### 4.3. Antimicrobial Assay

The antimicrobial activity of PCA was evaluated by examining the OD growth curves as follows. PCA dissolved in DMSO was added into 100 mL of beer extract broth obtain a final concentrations of 10, 20, 30 or 40 μg/mL, and the control culture was supplemented with 1% DMSO alone. The medium was inoculated with 100 μL of a freshly cultured suspension of *R. solanacearum* (1 × 10^9^ cfu/mL). The cultures were incubated at 180 r/min for 36 h at 30 °C, and cell growth was monitored spectrophotometrically (the optical density at 600 nm was recorded at 2 h intervals). All treatments were determined in triplicated and calculated to obtain an average value.

### 4.4. Bacterial Morphology

The morphology of *R. solanacearum* cells in the presence of PCA was evaluated using a scanning electron microscope. *R. solanacearum* at the logarithmic growth phase was diluted into a 10^8^ cfu/mL suspension of beer extract broth. PCA was added to the bacterial suspension to reach a final concentration of 30 μg/mL and 40 μg/mL. The bacterial suspension was then shaken at 180 r/min and 30 °C for 12 h. The cells were collected by centrifugation at 6000 rpm for 5 min, washed three times with 0.1 mol/L pH 7.0 phosphate buffer, and were then fixed in a 2.5% glutaraldehyde solution at 4 °C overnight. After fixation, the cells were dehydrated in a graded ethanol series (1 mL; 30%, 50%, 70%, 85% and 95%) with two changes every 5 min. The final cells were resuspended in Tert-butanol and fixed on the smooth surface of aluminum foil for observing after spraying with gold. Suspensions with 0.1% DMSO added and an untreated control were used.

### 4.5. Biofilm Formation Assay

The biofilm formation assay was performed by crystal violet staining as described by Petters *et al.* with slight modifications [53]. A polystyrene microtiter plate assay was used to quantify biofilm formation by *R. solanacearum*. Five centrifuge tubes were sterilized, and to each tube the 5 mL mixed cultures were added (5 μL of inoculum (OD_600_ ≈ 1.0) mixed with a final PCA concentration of 10 μg/mL, 20 μg/mL, 30 μg/mL and 40 μg/mL in beer extract broth and without PCA as a control). Biofilm growth was initiated by inoculating 200 μL of mixed cultures into individual wells of a 96-well microtiter plate. The plates were sealed with plastic wrap and incubated without shaking for 12, 24 and 36 h at 30 °C. At the end of the incubation period, the liquid medium was removed and immediately washed three times with distilled water. Each well was stained with 0.1% crystal violet for 30 min. After staining, each well was washed three times with distilled water to remove excess stain. The crystal violet was removed from the complex with 200 μL of 95% ethanol, and the absorbance values of biofilms was measured on a microplate reader at OD_490nm_. Each treatment had three replications, and the experiment was performed three times.

### 4.6. Swarming Assay

The swarming assay was based on Englert *et al.* [54] with slight modifications. A semisolid medium containing 0.35% agar [55] supplemented with PCA at various concentrations (10, 20, 30 and 40 μg/mL) was prepared. Then, the petri dishes were air-dried for 30 min on a bacteria-free work bench. An overnight culture of *R. solanacearum* (OD_600_ ≈ 0.8) was collected and washed twice using sterile water at 6000 rpm for 5 min and resuspended in sterile water. The bacterial suspension (2 μL) was drop-inoculated at the center of semisolid medium plates. The colony diameters were measured in both the vertical and the horizontal direction on each plate in triplicate after incubation for 24 h and 48 h at 28 °C. The results were expressed as the mean of three independent experiments.

### 4.7. Effect of PCA on Seedling Tobacco Incubated with R. solanacearum

Pot experiments were conducted to appraise the effect of PCA for controlling tobacco bacterial wilt. First, 5 mL of a freshly culture of a *R. solanacearum* inoculum solution at 10^7^ cfu/mL was inoculated in the rhizosphere. Then, a 10 mL solution of PCA (40 mg/L) or streptomycin (40 mg/L) was irrigated to the tobacco roots, which were inoculated with *R. solanacearum* two days later. A mixture consisting of DMSO with water in the proper proportion was poured on the control tobacco seedlings. The seedlings were cultivated in the greenhouse at a temperature of 30 ± 1 °C with a relative humidity of 85% to 90%. The incidence of bacterial wilt was monitored every two days, from seven days to 19 days after inoculation. In this experiment, each treatment had three replicates and each replicate included 20 tobacco plants. The disease rate was quantified as 0 = no symptoms; 1 = a small number of wilting leaves or a side with a streak spot; 2 = a diseased side with over half wilting leaves or a stem with a black steak spot under the top of the tobacco plant; 3 = a diseased side with over two-thirds wilting leaves or a stem with a black steak spot up to the top of the tobacco plant; and 4 = the entire plant died. To determine disease index and the control efficiency, we used the following formula:

(1) $$\text{Disease index}=\frac{\sum \left(n_{i}\times v_{i}\right)}{N\times 4}\times 100$$

where *n_i_* = the number of plants with respective disease index, *v_i_* = disease index (0, 1, 2, 3, 4), and *N* = the total number of plants used in each treatment.

(2) $$\text{Control efficiency (\%)}=\frac{CK-T}{CK}\times 100(\%)$$

where *T* = the disease index of treatment, *CK* = the disease index of control group.

## 5. Conclusions

In conclusion, this study evaluated the antibacterial activity of plant-derived protocatechualdehyde (PCA), which is the major active ingredient of *S. miltiorrhizae* against *R. solanacearum*. The results of this study indicate that the PCA had the strongest antibacterial activity and could be a potential antibacterial agent. The biological activity of PCA is largely due to its disruptive effect on the cell structure and shapes. Moreover, PCA could significantly reduce biofilm formation and suppress the swarming motility of *R. solanacearum*. Experiments conducted in the greenhouse showed that PCA also significantly reduced the incidence of bacterial wilt of tobacco. Overall, this is the first report concerning the antibacterial effects of PCA on plant-pathogenic bacteria. Further studies are expected to assess its control efficacy in the field and to explore its optimal application. Moreover, further structural modification and design based on PCA could improve the biological activity and contribute to the development of new antibacterial drugs against infections of *R. solanacearum*.
